# Co-Occurrence of Endometriosis with Systemic Lupus Erythematosus: Genetic Aspects

**DOI:** 10.3390/ijms26146841

**Published:** 2025-07-16

**Authors:** Maria I. Zervou, Theoni B. Tarlatzi, Grigoris F. Grimbizis, Timothy B. Niewold, Basil C. Tarlatzis, George Bertsias, George N. Goulielmos

**Affiliations:** 1 Section of Molecular Pathology and Human Genetics, Department of Internal Medicine, School of Medicine, University of Crete, 71003 Heraklion, Greece; mzervou@med.uoc.gr; 2 Unit for Human Reproduction, 1st Department of Obstetrics and Gynecology, School of Medicine, Aristotle University of Thessaloniki, 54124 Thessaloniki, Greece; nonika.tarlatzi@gmail.com (T.B.T.); grigoris.grimbizis@gmail.com (G.F.G.); basil.tarlatzis@gmail.com (B.C.T.); 3 Barbara Volcker Center for Women and Rheumatic Disease, New York, NY 10021, USA; niewoldt@hss.edu; 4 Hospital for Special Surgery, 535 East 70th Street, New York, NY 10021, USA; 5 Rheumatology and Clinical Immunology, School of Medicine, University of Crete, 71403 Heraklion, Greece; gbert@med.uoc.gr; 6 Institute of Molecular Biology and Biotechnology, FORTH, 70013 Heraklion, Greece; 7 Department of Internal Medicine, University Hospital of Heraklion, 71500 Heraklion, Greece

**Keywords:** systemic lupus erythematosus, endometriosis, inflammation, autoimmunity, genetics, gene polymorphisms

## Abstract

Previous studies have shown that patients with a history of endometriosis have an increased susceptibility for developing a big number of comorbidities, including various autoimmune diseases. Endometriosis is a complex, inflammatory, estrogen-dependent, heterogeneous gynecological disorder with an incidence of up to 10% in women of reproductive age. It is characterized by the implantation and growth of endometrial tissue outside the uterus and is associated with dysmenorrhea, deep dyspareunia, pelvic pain and infertility. Systemic lupus erythematosus (SLE) is a chronic, heterogeneous autoimmune disorder of the connective tissue, characterized by impaired innate and adaptive immune responses and the production of pathogenic autoantibodies that drive inflammation and damage in multiple organs. Its etiology is elusive yet associated with high heritability. Importantly, it has been found that endometriosis and SLE share some underlying molecular and cellular pathways. In the present study, we sought to delineate the co-occurrence of endometriosis with SLE from the biological and genetic viewpoint, aiming to identify the putative shared genetic components and clarify the underlying pathogenetic mechanisms. This information may contribute further to the design of new therapeutic protocols for both disorders under study.

## 1. Introduction

Endometriosis is an enigmatic, common, benign, multifactorial gynecological disorder that affects 8–10% of reproductive age women across the globe and affecting seriously the quality of life [1]. Many genetic and epigenetic factors are combined with environmental ones, thus leading to this disease [2]. Endometriosis is defined by the growth of endometrial tissue outside the uterine cavity. It is associated with chronic pelvic pain, dysmenorrhea, dyspareunia, subfertility and infertility [3]. In women with endometriosis, various changes in immunity mediated by T cells may facilitate the implantation of endometrial fragments or cells ectopically [3]. Importantly, the immune system’s deregulation influences endometriosis, which shares many similarities with autoimmune diseases. Thus, endometriosis is often associated with the presence of a large variety of antibodies in the blood and peritoneal fluid of patients, including antinuclear (ANA), antiphospholipid (aPL), antithyroid, anti-steroid, anti-survivin, anti-laminin-1, anti-carbonic anhydrase, anti-α-enolase, anti-DNA autoantibodies, as well as autoantibodies to endometrial antigens (AEA), while it is also characterized by the aberrant function of immune cells and elevated levels of inflammatory cytokines [4,5,6].

Systemic lupus erythematosus (SLE) is a chronic, severe, multiorgan systemic autoimmune disease that predominantly affects women, which is characterized by a complex genetic inheritance and a wide spectrum of clinical manifestations that may involve different organs and tissues [3]. SLE patients appear high titers of autoantibodies directed against serum proteins as well as native cell surface and nuclear components, due to an impaired clearance of the apoptotic cells and the loss of B cell tolerance [7]. Interactions between susceptibility genes and environmental factors lead to the disease’s development [8].

Various studies have shown that women with endometriosis have a higher susceptibility for developing various chronic diseases as comorbidities, including asthma [9,10], cancer [9] and cardiovascular diseases [11]. Of note, a remarkable number of autoimmune diseases co-occurring with endometriosis has been detected, including ankylosing spondylitis (AS) [12], coeliac disease (CeD) [13], Crohn’s disease (CD) and ulcerative colitis (UC) [14], multiple sclerosis (MS) [9], psoriasis (PS) and psoriatic arthritis (PsA) [15], rheumatoid arthritis (RA) [9], Sjogren’s syndrome (SS) [9,16], systemic lupus erythematosus (SLE) [9,17] and autoimmune thyroid disorder [18]. It is worth noting that it is not yet known whether autoimmune diseases represent a risk factor of endometriosis or endometriosis and autoimmune disorders just share mediators involved in their pathogenesis. Moreover, it is still unclear whether some characteristics of endometriosis, i.e., aberrant immunological response and chronic inflammation, may increase the long-term risk of developing another autoimmune disease.

Recently, we attempted to delineate the genetic basis of the co-occurrence of endometriosis with various autoimmune diseases, including RA [5], AS [6] and SS [19]. In this article, we present an overview of the gene polymorphisms known so far to be associated with an increased risk for developing both endometriosis and SLE, aiming to clarify the causal association between endometriosis and SLE, and give insights about the functional and pathophysiological role of these shared polymorphisms.

## 2. Genetics of Endometriosis and SLE

**Figure 1:**
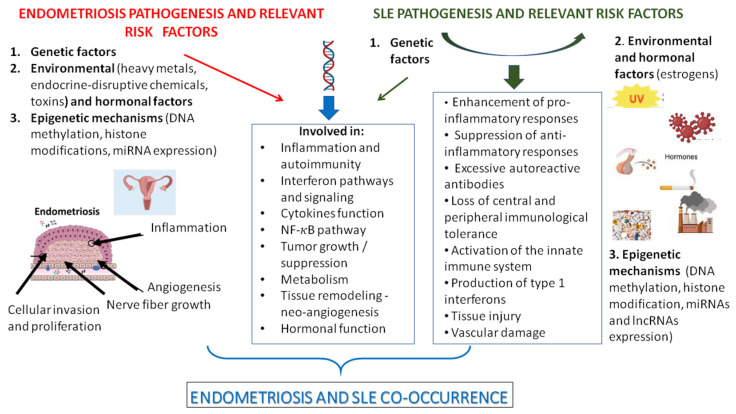
Proposed pathogenetic mechanisms for endometriosis–SLE co-occurrence. Genetic risk loci associated with both diseases are classified into major distinct mechanistic pathways. Shared genetic risk loci are associated with inflammation and autoimmunity, interferon pathways and signaling, cytokines function, NF-*κ*B pathway, tumor growth/suppression, metabolism, tissue remodeling and neo-angiogenesis as well as hormonal function. All pathways, apart from the genetic factors, are influenced also by epigenetic and environmental factors, thus formulating the cross-link between the shared genetic background and the diseases under investigation. Figure partially adapted from Zervou et al., 2024 [22].

Studies on Australian twins and monozygotic twins [19,20] initially suggested a genetic contribution to endometriosis. Nowadays, a big number of endometriosis-associated single nucleotide polymorphisms (SNPs) of moderate effects were revealed through gene association studies [21,22] and genome-wide association studies (GWAS), including the SNPs of Wnt family member 4 (*WNT4*), the antisense non-coding RNA in the INK4 locus (*ANRIL*, also known as *CDKN2B-AS1*), the inhibitor of DNA binding 4 (ID4), growth-regulating estrogen receptor binding 1 (*GREB1*), vezatin adherens junctions transmembrane protein (*VEZT*), fibronectin 1 (*FN1*), spectrin repeat containing nuclear envelope protein 1 (*SYNE1*), coiled-coil domain containing 17 (*CCDC17*), follicle stimulating hormone subunit beta (*FSHB*), estrogen receptor 1 (*ESR1*), UDP glucuronosyltransferase family 2 member B28 (*UGT2B28*), ubiquitin specific peptidase 17 like family member 2 (*USP17L2*), interleukin 16 (*IL-16*), potassium channel tetramerization domain containing 9 (*KCTD9*), ARF Like GTPase 14 effector protein (*ARL14EP*), long intergenic non-protein coding RNA 629 (*LINC00629*), homeobox A10 (*HOXA10*), PDZ and LIM domain protein 5 (*PDLIM5*), Bcl2 modifying factor (*BMF*), actin-like 9 (*ACTL9*) and bassoon presynaptic cytomatrix protein (*BSN*) gene loci [23,24,25,26]. The endometriosis-associated SNPs have been categorized according to the function of the respective encoded proteins, which are involved in cell cycle regulation, cell adhesion, differentiation, proliferation, inflammation, sex steroid receptors pathways, matrix remodeling, immunity, vascular function, angiogenesis and oxidative stress [24,25] [Figure 1]. Epigenetic aberrations have been reported to play a crucial role in both pathogenesis and development of specific manifestations of endometriosis. Particularly, differential levels of DNA methylation and microRNA expression, as well as histone modifications, reflect the repertoire of the epigenetic modifications at the cellular level [19,27,28].

SLE results from the interaction of both genetic and environmental factors. To date, more than 100 SLE- associated risk loci have been identified mainly through GWA studies, including among others members of the major histocompatibility complex major histocompatibility complex (*MHC*) [29], signal transducer and activator of transcription 4 (*STAT4*) [30], neutrophil cytosolic factor 2 (*NCF2*), IKAROS family zinc finger 1 (*IKZF1*), interferon regulatory factor 8 (*IRF8*), interferon-induced helicase C domain-containing protein 1 (*IFIH1*), tyrosine kinase 2 (*TYK2*) [31], PHD and ring finger domains 1 (*KIAA1542*), integrin subunit alpha M (*ITGAM*), PX Domain Containing Serine/Threonine Kinase Like (*PXK*), interferon regulatory factor 5 (*IRF5*), Deoxyribonuclease 1L3 (*DNASE1L3*), protein tyrosine phosphatase, receptor type, C (PTPRC), janus kinase 2 (*JAK2*), I-kappa-B kinase epsilon (*IKBKE*), broad complex-tramtrack-bric a brac and Cap’n’collar homology 2 (*BACH2*), ataxin 1 (*ATXN1*) [32], sprouty-related EVH1 domain-containing protein 2 (*SPRED2*), Transcription factor 7/S-phase kinase-associated protein 1 (*TCF7/SKP1*), Fcgamma Receptor IIa (*FCGR2A*), IKAROS family zinc finger 2 (*IKZF2*), IKAROS family zinc finger 2 (*IKZF3*), phospholipase D2 (*PLD2*), SH2B adapter protein 3 (*SH2B3*), solute carrier family 15 member 4 (*SLC15A4*), ubiquitin-conjugating enzyme E2 L3 (*UBE2L3*), interferon inducible X-linked gene CXorf21 (*CXorf21*), interferon regulatory factor 7 (*IRF7*), CD44 (*CD44*), juxtaposed with another zinc finger protein 1 (*JAZF1*), B-lymphoid tyrosine kinase (*BLK*) and AT-rich interactive domain-containing protein 5B (*ARID5B*) [26] SNPs. These loci explain only ~30% of the disease’s heritability [33] and have been associated with immune response and inflammation, transcription mechanisms, neo-angiogenesis, lymphocyte activation and proliferation [Figure 1], while a pathway enrichment analysis in meta-analysis GWAS data pinpointed the role of various signaling pathways in the development of SLE, including the B-cell receptor, IL-4 and CTLA4/T cell co-stimulation and activation [34]. Epigenetic deregulation was found to occur generally in SLE [35]. In this framework, decreased levels of DNA methyltransferase 1 (Dnmt1) result in the hypomethylation of the DNA of CD4+ T cells in SLE, thus leading to an autoreactivity of these cells and the subsequent production of various autoantibodies, proinflammatory chemokines and cytokines [36]. Notably, microRNAS (miRs) and long non-coding RNAs (lncRNAs), which play an important role in epigenetic processes, have been also suggested to be involved in the development of SLE [37].

## 3. Shared Susceptibility Loci Between Endometriosis and SLE

In the framework of the current research, we investigated a potential shared genetic background regarding the co-occurrence of endometriosis and SLE. To our knowledge, it is the first attempt in the literature focusing on the genetic basis of the subsequent development of SLE in patients with endometriosis thus far. The method used for this article included case-control and GWAS, systematic reviews and cross-sectional studies. Notably, case reports/series, editorial letters, expert opinions, and conference abstracts were excluded. The papers included were written in English language and published from 2000 to 2025. Five electronic databases were used (PubMed, PubMed Central (PMC), Google Scholar, Web of Science and MEDLINE). The keywords that were included in the search study were endometriosis, systemic lupus erythematosus, genetics, gene polymorphisms, association studies, GWAS, whole exome sequencing (WES). Two authors have worked individually to extract the data following the specified criteria and no disagreements arose during the process.

An association between endometriosis and a higher risk of SLE has been reported in previous studies [9,17]. Thus, we attempted to search for shared genetic factors involved in the co-occurrence of these diseases, as well as for the corresponding shared pathogenetic mechanisms underlying both conditions. Our literature research showed that the *ESR1* (or *ER-alpha*) rs9340799 [38,39] and rs2234693 [40,41], Fc receptor-like 3 (*FCRL3*) rs7528684 [42,43], forkhead box protein 3 (*FOXP3*) rs3761549 [42,44], interleukin-6 (*IL-6*) rs18001796 [45,46], interleukin-10 (*IL-10*) rs1800871 [47,48] and rs1800896 [49,50], interleukin-12B (*IL-12B*) rs17860508 [51,52], *IL-16* rs11556218 [22,53], *IRF5* rs10488631 [54,55], methylenetetrahydrofolate reductase (*MTHFR*) rs1801133 [56,57], nuclear factor kappa-light-chain-enhancer of activated B cells (*NF-kB*) rs28362491 [58,59], protein tyrosine phosphatase, non-receptor type 2 (*PTPN22*) rs2476601 [60,61], *STAT4* rs7574865 [30,62] and rs7582694 [63,64], tumor necrosis factor-alpha (*TNF-a*) rs1800629 [65,66] and tumor suppressor protein p53 (*TP53*) rs1042522 [67,68] SNPs are associated with both diseases under study (Table 1).

Interestingly, in a previous bioinformatics study conducted by Joseph and Mahale [69], which led to the development of a database called “Endometriosis Knowledgebase”, two common pathways enriched in endometriosis- and SLE-associated genes were detected, referred to the apoptosis signaling and the inflammation-mediated chemokine/cytokine pathways, respectively.

## 4. Biological Mechanisms Related to the Co-Occurrence of Endometriosis and SLE

### 4.1. Polymorphisms in Genes Associated with Interferon Pathways and Signaling

STAT4 protein, a member of a family of latent cytosolic transcription factors, is encoded by *STAT4* gene, which plays a central role in IFN signalling and is involved in the regulation of various immunological processes [70]. Both *STAT4* rs7574865 [30,62] and rs7582694 [63] SNPs have been associated with an increased risk of both endometriosis and SLE. The TT genotype of *STAT4* rs7574865 G/T polymorphism was found to be more frequent in women with minimal or mild endometriosis compared to the controls [62]. Importantly, it has been suggested that rs7574865 SNP may impair either the gene expression or mRNA splicing of *STAT4* gene, thus being enrolled in IFN signaling [62]. This biological process may strengthen the involvement of rs7574865 SNP in endometriosis, considering that a combination of clinical data and increased levels of certain cytokines has suggested a strong relationship between T-helper 1 (Th1) response pattern and deep infiltrating endometriosis [71]. Furthermore, the role of STAT4 regarding the optimal differentiation of T-helper 17 (Th17) cells has been demonstrated [30], with these cells contributing to the development of chronic inflammatory diseases, including SLE [72].

An association of the “C” allele of the *STAT4* rs7582694 G > C SNP with the development of SLE and the occurrence of some clinical manifestations of the disease, including neurologic ones and autoantibodies production, has been confirmed [64]. A similar significant association between “C” allele and endometriosis was detected [63], while an association of this SNP with clinical manifestations including dysmenorrhea, chronic pelvic pain and dyspareunia was revealed as well [63]. Moreover, it has been assumed that both SNPs may affect the gene expression and mRNA splicing of *STAT4* gene and, therefore, play a role in the induction of strong Th1 and Th17 cytokine responses [5,63].

Interferon regulatory factor 5 (IRF5) is a multifunctional transcription factor encoded by the IRF5 gene, which is involved in the transcriptional activation of various proinflammatory cytokines and type 1 interferon (IFN)-related genes [73]. The minor allele “C” of rs10488631 SNP of this gene was found to be strongly associated with a risk for SLE as well as elevated levels of circulating type 1 IFN in SLE patients [54]. The same allele was shown to be associated with an increased risk of moderate/severe endometriosis development [55]. Considering that IRF5 contributes to the formation of type I IFN and pro-inflammatory cytokines, a pathogenetic role of this protein for endometriosis can be suggested.

### 4.2. Polymorphisms in Inflammation and Autoimmunity-Related Genes

TNF-α gene encodes the TNF-α cytokine, which is a major regulator of autoimmunity and inflammation and is involved in T cell response, neutrophil activation and increase of expression levels of adhesion molecules [74]. Increased levels of TNF-α in the serum of SLE patients compared to healthy controls have been detected and correlated with active disease [75]. Moreover, TNF-α is a pro-inflammatory cytokine involved in the development of the ectopic endometrial tissue in the peritoneal cavity and elevated levels of the circulated protein have been correlated with the severity of endometriosis [64]. The presence of minor allele “A” of rs1800629 SNP of *TNF-α* gene was found to enhance the transcription levels of TNF-α, compared with allele “G”, thus resulting in increased protein levels in serum [76]. This allele was reported to modify the consensus sequence (5′-GCCNNNGGC-3′) for a binding site of the transcription factor AP-2 [77]. Notably, taking into account that increased levels of TNF-α facilitate the implantation of ectopic endometrial tissue [76], this protein can be established as a factor related to the development of endometriosis. Moreover, it has been reported that “A” allele of rs1800629 SNP is associated with an increased susceptibility to SLE [77], due to the elevated levels of TNF-α protein, which contribute to the development of SLE [66].

The non-receptor type 2 *PTPN22* gene, coding for the lymphoid-specific phosphatase Lyp, functions as a potent down-regulator of T-cell activation [78]. The “T” allele of the *PTPN22* functional rs2476601 (C1885T) results in the production of a gain-of-function Lyp enzyme, the physical interaction between Lyp and Csk proteins is disrupted and T-cell activation cannot be suppressed [79]. It has been reported that, in SLE, 1858T variant is associated with elevated levels of inflammatory cytokines [80]. In endometriosis, allele “T” appears at a higher frequency in women with moderate/severe stage of endometriosis than in patients with a minimal/mild stage [60].

The *FOXP3* gene, a member of the Forkhead/Winged-helix family, is an X-linked gene encoding the Forkhead box P3 (FOXP3) transcription factor, whose function is related to the regulation of T cell activation as well as the development and function of T regulatory (Tregs) cells [81]. The *FOXP3* rs3761549 (−2383C > T) SNP was shown to be involved in the alteration of the sequence of the binding site of the transcription factor Ying Yang 1 (YY1) of the *FOXP3* gene, thus affecting the expression levels of the gene [82]. Interestingly, YY1 protein inhibits differentiation and function of regulatory T cells by blocking FOXP3-induced expression and activity of target genes by physically binding and blocking FOXP3 [82]. Therefore, endometriosis may arise through the highly disturbed function of Tregs, considering their crucial role in the regulation and suppression of immune response [42].

### 4.3. Polymorphisms in Cytokine Genes

The IL-6 gene codes for a multifunctional proinflammatory cytokine that links the endocrine and the immune systems [83]. It is involved in immune responses, while alterations in the expression levels or other changes may lead to abnormal cellular and humoral immune responses. The rs1800796 (-572G/C) *IL-6* SNP may influence IL-6 transcription levels both in vitro and in vivo [84], given that allele “G” is located on a predicted transcription factor binding site [85]. Increased amounts of IL-6 have been detected in serum as well as in peritoneal fluid of endometriosis patients [86]. Moreover, both mRNA and protein IL-6 levels were found to be elevated in serum and peripheral blood mononuclear cells in SLE patients, while IL-6 blockade may reduce autoantibody production and abrogate disease activity in SLE patients [87].

IL-10, produced by T-helper 2 (Th2) cells and macrophages, inhibits the synthesis of pro-inflammatory cytokines in activated macrophages and T lymphocytes [88]. The rs1800871 (-819C/T) SNP, located within the functional promoter of *IL-10* gene, was found to affect in endometriosis both the mRNA and the protein levels of IL-10 [89], with women of the TT genotype showing a 2-fold increased risk for developing endometriosis compared to “C” allele carriers. Given that allele “C” has been associated with higher levels of IL-10 compared with the “T” allele in patients [47], it can be assumed that this is a mechanism that exerts its function by down-regulating the peritoneal cavity cell inflammation [47]. Similarly, this SNP was also associated in SLE with an increased disease activity, considering that SLEDAI score was found to be higher in TT patients [56]. Furthermore, it has been found that “G” allele of *IL-10* rs1800896 (G/A at position −1082) SNP is associated with increased susceptibility to both SLE and endometriosis [49,50,90].

IL-12 is a proinflammatory protein that induces the production of interferon-γ (IFN-α) and is related to the differentiation of naïve T-cells into T-helper (Th) 1 and Th17 cells [91]. The *IL-12B* rs17860508 (-6415 CTCTAA/GC) variant, associated with a risk for developing endometriosis and SLE [51,52], is located in a putative binding site for transcriptional factor specificity protein 1 (Sp-1) [92] and has been associated with alterations at the gene expression levels and mRNA stability, with the GC allele in *IL-12B* promoter exhibiting stronger activity upon binding of Sp1 and leading finally to higher cytokine production [93]. Zhao et al. [51] showed that patients carrying the GC allele had an increased susceptibility for ovarian endometriosis compared to individuals carrying the CTCTAA/CTCTAA genotype. Furthermore, an increased frequency of GC allele of this SNP was found in patients with SLE [52].

Interleukin-16 (IL-16) is a T-cell proinflammatory cytokine that is secreted by CD8+ T-lymphocytes, mast cells and B-cells [22]. The *IL-16* rs11556218 SNP corresponds to a missense mutation wherein asparagine (Asn) is substituted by lysine (Lys) and allele ‘G’ is associated with an increased susceptibility of developing both endometriosis [22] and SLE [53]. Allele “G” has been hypothesized to result in an increased transcription activity of *IL-16* gene, thus leading to the production of elevated levels of IL-16 protein in serum [22]. Notably, elevated levels of this protein have been also observed in peritoneal fluid of women with endometriosis [94].

### 4.4. Polymorphisms in Genes Involved in the NF-κB Pathways

Nuclear factor-kB (NF-kB) is a pivotal transcriptional factor that, upon activation, plays an important role in the promotion of immune and inflammatory response and the regulation of cell proliferation and/or transformation, anti-apoptosis, cell invasion and angiogenesis [95]. A common insertion/deletion polymorphism (−94 insertion/deletion ATTG, rs28362491), located between two putative key promoter regulatory elements of the *NF-kB* gene, has been positively associated with moderate/severe endometriosis [58]. It has been reported that activation of NFKB1, resulting in the production of elevated levels of proinflammatory cytokines and chemokines, leads to the early development of endometriotic lesions [96]. Cen et al. [59] showed an association of rs28362491 polymorphism with SLE and a gene-to-gene interaction between *NFKB1* rs28362491 and *TNIP1* rs3792783 SNPs, thus indicating their synergistic contribution to the increased predisposition of SLE [59].

The *FCRL3* gene encodes a glycoprotein that mediates both B cell receptor signaling and plasma B cell maturation and antibody production [97]. The “C” allele of the *FCRL3* rs7528684 (-169C/T) SNP has been correlated with a higher promoter activity and increased expression levels of the gene, thus being more avidly bound by NF-kB and suggesting a direct functional role for this allele in SLE [4,43]. In endometriosis, the increased FCRL3 expression in B cells may affect normal B cell functions, resulting in the appearance of pelvic pain, dyspareunia and infertility [42].

### 4.5. Polymorphisms in Tumor Growth/Suppression and Metabolism-Related Genes

The *TP53* gene, a tumor suppressor gene, encodes the TP53 nuclear phosphoprotein, which is a transcription factor involved in many essential functions, including programmed cell death, cell cycle regulation and DNA repair [98]. The rs1042522 (G/C) SNP, located within exon 4 (codon 72) is a functional polymorphism leading to an arginine to proline substitution. It is located in the proline-rich domain of p53, which is important for the initiation of the programmed cell death. It has been shown that Arg72Pro substitution influences the protein’s function [68]. The SLE risk variant Pro72 may lead to autoimmunity through the increased apoptosis and impaired clearance functions for dying cells and the attenuation of the deletion of autoreactive lymphocytes [99]. In the same framework, it has been reported that persisting clearance defects are very closely associated with the development of chronic autoimmunity [99]. Furthermore, women carrying the Pro72 variant appear an increased risk for endometriosis [100].

The *MTHFR* gene encodes MTHFR protein, which represents a key regulatory enzyme in folate and homocysteine metabolism, playing an important role in methylation that regulates crucially DNA synthesis, repair, and gene expression [101]. Rs1801133 (C677T; Ala222Val) SNP of *MTHFR* gene was shown to influence the enzyme activity [102]. The endometriosis-risk allele “T” [56], leading to the Ala/Val substitution, results in elevated plasma homocysteine levels, which in combination with vascular inflammation may lead to the development of cardiovascular disease, a known comorbidity of endometriosis [11]. Moreover, carriers of “T” allele have been associated with an increased risk for SLE compared to “C” allele [57].

### 4.6. Polymorphisms in Genes Involved in Hormonal Function

The *ESR1* gene encodes a steroid nuclear ligand that is considered as the primary receptor for estrogen [103]. Several polymorphisms of this gene have been identified, which may influence the impact of estrogen, thus leading to clinically relevant phenotypes [103]. Rs9340799 SNP involving an A-to-G transition, also known as the -351A > G polymorphism, corresponds to an *Xba*I restriction site-based polymorphism. Carriers of allele “G” have an increased risk for endometriosis [38]. Interestingly, this allele was suggested that might disturb transcription efficiency, thus affecting the gene expression levels and estrogen-related molecular mechanisms [103,104]. Both rs2234693 (*Pvu*II) and rs9340799 (*Xba*I) ESR1 polymorphisms have been associated with an increased susceptibility for SLE, with the *Pvu*II “C” and *Xba*I “G” alleles conferring approximately a two to three-fold increase in risk for the disease [39]. Considering that both endogenous estrogen levels and exogenous estrogen exposure predispose for SLE [105], it has been suggested that any genetic variation resulting in an enhancement of the estrogen and estrogen receptors’ activities may increase the risk of SLE due to the important role of estrogens in the pathogenesis of the disease [39].

## 5. Discussion

Endometriosis is a disease characterized by both inflammatory responses and immune system dysregulation. The presence of various autoantibodies in blood and peritoneal fluids of patients, the high prevalence of several autoimmune diseases appearing as co-existing conditions, the remarkable number of autoimmunity-associated genetic polymorphisms observed in women with endometriosis, the chronicity of the disease and the raising interest and clinical studies regarding the beneficial effects of immunomodulatory agents on endometriosis’ patients, have strengthened the hypothesis that endometriosis resembles an autoimmune disease [2,3,106]. Furthermore, the origin of ectopic endometrial tissues has been associated with immune systems abnormalities and a deficient cellular immunity in patients [107].

The data of the current study pointed out a link between endometriosis and SLE from the genetic and molecular biology point of view, thus providing some explanations for this co-occurrence. Due to the limited number of relevant cohort studies [9,17,18], the association between these disorders remains obscure apart from our present study focusing on the genetic components of these diseases. However, in an attempt the causal association between endometriosis and SLE to be clarified in depth, some suggestions can be considered. In this context, the disturbance of the immune system was suggested to explain the correlation between both conditions under study [108]. There are several pieces of evidence emphasizing the significant role of the immune dysregulation and the overactive adaptive immune system in the development of both diseases through a decreased apoptosis rate of neutrophils and dysfunction of the innate and adaptive immune system [18]. Moreover, women with endometriosis have shown a decline in natural killer cell cytotoxicity and elevation in both the number and activation of macrophages. Immune dysregulation and imbalanced hormonal milieu result in excessive endometrial tissue deposition into ectopic sites [2]. Further dysregulation in the immune system leads to the development of autoimmune disease. In the same context, alterations in expression levels of estrogen and estrogen receptors contribute to the development of both conditions [3,109]. Of note, apart from the identified genetic risk factors, the endometrial cells can be disseminated and expressed through the uterine tubes, because immune deregulation and imbalanced hormonal milieu result in excessive endometrial tissue deposition into ectopic sites [3,25].

Importantly, although a high number of SLE- and endometriosis-associated SNPs have been detected so far, the functional significance of a small number only of these genetic associations has been elucidated, thus weakening their potential for further translation into therapeutic interventions through the discovery of putative new therapeutic targets. Previous studies have reported a significant increase in the risk of incident SLE in women with endometriosis compared to unaffected controls, ranging from 1.36 to 2.73-fold [17,18,110], whereas women with SLE have a significantly higher risk of endometriosis compared with women without SLE (adjusted hazard ratio 1.32, 95% CI: 1.02–1.70) [111]. Therefore, clinicians should always be alerted for the possible co-occurrence of endometriosis with SLE. In parallel, women with endometriosis have to be alerted that if they appear any symptoms characterizing SLE, such as problems in skin, joints, kidneys, heart or blood vessels [109], they should report it to a rheumatologist in order a suitable medication to be provided. Importantly, appropriate dietary supplements that increase serum iron and selenium levels may reduce the risk of SLE [112].

Studies in SLE have emphasized the role of autoimmunity, proliferation and activation of immune cells, inflammation, vascular damage and tissue injury in the disease’s development [8,23] (Figure 1). Interestingly, by combining the genetic data presented in this article with previous ones referred to the genetic basis of the co-occurrence of endometriosis with RA, AS or SS [5,6,19], we conclude that seven SNPs are associated with endometriosis and SLE only. Particularly, *TP53* rs1042522, *NF-kB* rs28362491, *IL-10* rs1800896, *IL-16* rs11556218 and *FOXP3* rs3761549, as well as *ESR1* rs9340799 and rs2234693 SNPs, are not associated with the co-occurrence of endometriosis and RA, AS or SS. In this framework, the exploration of the shared genetics underlying SLE and endometriosis has led to important findings in the field of translational medicine. Table 1 presents the association of *IL-6* rs1800796 SNP with an increased susceptibility to both diseases under investigation [45,46], while an association between elevated levels of the serum IL-6 and SLE has been demonstrated as well [113]. IL-6 blockade may reduce autoantibody production and abrogate disease activity in SLE patients [114]. Tocilizumab, a humanized monoclonal antibody against the α-chain of the IL-6 receptor, has been considered as a good choice for the treatment of endometriosis [115]. Tocilizumab can also reduce lymphocyte activation and restore the homeostasis of B- and T- cells in SLE patients [116]. Moreover, *NF-kB* rs28362491 SNP is associated with an increased risk for both diseases [58,59]. Considering that NF-kB transcriptional activity results in the modulation of cell processes that are involved in the initiation and progression of endometriosis, the inhibition of NF-kB seems to be an efficient and promising therapeutic approach for this health condition as well [107]. Importantly, medication for women with endometriosis uses NF-kB inhibitors that suppress in vitro the proliferation of endometriotic cells [117]. Similarly, the inhibition of NF-κB-inducing kinase (NIK) represents a potential therapeutic approach for SLE, given that it leads to the inhibition of multiple pathways known to be involved in SLE and results in the improvement of various parameters associated with the disease, including survival rate, kidney function and lower proteinuria scores [118].

Recent data suggest that screening for autoimmune antibody panels could be considered in patients diagnosed with endometriosis. Accordingly, screening for autoimmune antibody panels targeting endometrial antigens, may be considered in women patients diagnosed with endometriosis, especially those experiencing infertility or severe disease of stage IV [119]. In the same framework, a panel of six autoimmune biomarkers has been suggested to be useful in setting up of noninvasive diagnostic test for detection of minimal-mild endometriosis [120]. Furthermore, it is known that endometriosis is associated with autoantibodies to endometrial antigens, α-enolase, steroid and gonadotropic hormones, while a wider spectrum of antibodies is detected in ovarian endometrioma (OEM) than in deep infiltrating endometriosis (DIE). It has been suggested that these antibodies have a high diagnostic value for OEM and DIE, and potential pathogenetic significance for endometriosis and associated infertility [4].

A weakness of our study to be considered refers to the lack of replication studies in the literature thus far, concerning the co-occurrence of endometriosis and SLE in patients of different racial and/or ethnic origin. This is an important issue, taking into account a differential role of the various polymorphisms involved in the development of these disorders, which depends on the ancestral backgrounds of the populations under examination [121,122]. Obviously, an in-depth analysis of the shared gene polymorphisms should be a next step in an attempt to clarify their functional significance and highlight the underlying molecular pathways. However, the prior performance of replication experiments and the confirmation of the polymorphisms associated with both diseases is a prerequisite. Thus, the potential clinical application of the genetic data associated with endometriosis and SLE for both precision medicine and therapeutic target discovery remains challenging, considering its potential in prioritizing causal genes of these diseases for further pharmaceutical intervention. Notably, the source of the inter-individual susceptibility to endometriosis or SLE does not lie exclusively in genetics, considering that accumulated information suggests that epigenetics mechanisms participate also in the susceptibility to these diseases [3,28]. The role of potential shared epigenetic factors is planned to be investigated in a future study.

In conclusion, the total information derived from the present article may deepen our understanding of the underlying cellular and molecular mechanisms and contribute to the design of new therapeutic protocols for endometriosis and SLE. However, further translational medical investigations are needed to clarify the link and mechanisms between both disorders under study. Clinical implications primarily involve the need for increased awareness and vigilance. Altogether, integrating clinical and molecular data using high-throughput technologies in combination with the employment of computational and bioinformatics tools, can confer to the development of biomarkers for early detection of the two-diseases’ coexistence and probably advance precision medicine for these women. The shared genetic basis opens up opportunities for developing new treatments or repurposing therapies across these conditions.

## Figures and Tables

**Table 1:** An overview of the genetic polymorphisms associated with the development of both endometriosis and SLE, as they have been confirmed by gene association studies and/or genome-wide association studies. SNPs that are associated with endometriosis and SLE only but not with other autoimmune diseases, i.e., RA, AS or SS are presented in bold.

dbSNP ID	Endometriosis and SLE-Associated Gene	Function	Association with Endometriosis	Association with SLE	References
**rs9340799** **rs2234693**	*ESR1*	An estrogen receptor and ligand-activated transcription factor	Allele “G”, OR 2.54 Allele “T”, OR = 1.53 (95% CI: 1.05–2.21; *p* = 0.025)	XX vs. xx, OR: 3.4 (95% CI: 1.1–10.5) PP vs. pp, OR: 3.1 (95% CI: 1.1–9.3)	[38,39,40,41]
rs7528684	*FCRL3*	An Ig receptor, mediating plasma B cell maturation and antibody production	Allele “C”, OR = 1.58 (95% CI: 1.17–2.12; *p* = 0.03)	Allele “C”, OR = 1.49 (95% CI: 1.16–1.92; *p* = 0.0017)	[42,43]
**rs3761549**	*FOXP3*	A regulator of T cell activation; down regulates cytokine production in T cells	Allele “T”, OR = 2.05 (95% CI: 1.22–3.45); *p* = 0.08)	Allele “T”, OR = 2.2 (95% CI: 1.4–3.3; *p* < 0.007)	[42,44]
rs1800796	*IL-6*	A pro-inflammatory cytokine; stimulator of osteoclast formation	Allele “C”, OR = 2.17 (*p* < 0.001)	Allele “C”, OR = 1.49 (95% CI: 1.10–2.01, *p* = 0.009	[45,46]
rs1800871 **rs1800896**	*IL-10*	An anti-inflammatory cytokine; inhibitor of Th1 differentiation	TT genotype, OR = 0.52 (*p* = 0.006) GG genotype, OR = 2.22 (95% CI: 1.25–3.94, *p* = 0.009)	Allele “T”, OR = 1.47 (95% CI: 1.12–1.94, *p* < 0.05) GG genotype, OR = 2.65 (95% CI: 1.21–5.82, *p* = 0.046)	[47,48,49,50]
rs17860508	IL-12B	A cytokine acting on T and natural killer cells	Allele “GC”, OR = 1.25 (95% CI: 1.09–1.44, *p* = 0.01)	Allele “GC”, *p* < 0.001	[51,52]
**rs11556218**	*IL* *-16*	A pleiotropic pro-inflammatory cytokine	Allele “G”, OR = 3.02 (95% CI: 2.17–4.20, *p* < 0.0001)	Allele “G”, OR = 2.25 (95% CI: 1.64–3.13, *p* < 0.001)	[22,53]
rs10488631	*IRF5*	A pleiotropic transcription factor involved in virus-mediated activation of IFN	Allele “C”, OR = 1.79 (95% CI: 1.09–2.94, *p* = 0.028)	Allele “C”, OR = 0.54 (95% CI: 0.37–0.79, *p* = 0.0012)	[54,55]
rs1801133	*MTHFR*	A key regulatory enzyme in folate and homocysteine metabolism	Allele “T”, OR = 1.899 (95% CI: 1.076–3.318, *p* = 0.0269)	Allele “T”, OR = 1.766 (95% CI: 1.014–3.075, *p* = 0.04)	[56,57]
**rs28362491**	*NF-kB*	A major transcription factor of genes involved in both the innate and adaptive immunity	−94 insertion/ deletion ATTG polymorphism, OR = 1.968 (95% CI: 1.442–2.686, *p* < 0.0001)	−94 insertion/ deletion ATTG polymorphism, OR = 1.14 (95% CI: 1.00–1.31, *p* = 0.047)	[58,59]
rs2476601	*PTPN22*	A lymphoid-specific phosphatase; down-regulator of T cell activation	Allele “T”, OR = 2.05 (95% CI: 1.28–3.29, *p* = 0.004)	Allele “T”, OR = 1.91 (95% CI: 1.11–3.90, *p* = 0.017)	[60,61]
rs7574865 rs7582694	*STAT4*	A transcription factor involved in Th17 differentiation and monocyte activation	TT genotype, OR = 1.03 (95% CI: 0.68–1.58, *p* = 0.047) Allele “C”, OR = 1.986 (95% CI: 1.262–3.126, *p* = 0.002)	Allele “T’, OR = 1.55 (95% CI: 1.34–1.79, *p* = 1.87 × 10^−9^) Allele “C”. OR = 1.539 (95% CI: 1.209–1.969, *p* = 0.0004)	[30,62,63,64]
rs1800629	*TNF-α*	A multifunctional pro-inflammatory cytokine	Allele “A”, OR = 3.4 (95% CI: 1.25 = 9.23, *p* = 0.029)	Allele “A”, OR = 1.78 (95% CI: 1.45–2.19, *p* < 0.001)	[65,66]
**rs1042522**	*TP53*	A tumor suppressor protein	Allele “G”, OR = 1.32 (95% CI: 1.14–1.53, *p* < 0.001)	Allele “G”, OR = 0.89 (95% CI: 0.81–0.97, *p* = 0.01)	[67,68]

## Abbreviations

The following abbreviations are used in this manuscript:

AEA	Autoantibodies to endometrial antigens
ANA	Antinuclear antibodies
AS	Ankylosing spondylitis
aPL	Antiphospholipid antibodies
CeD	Coeliac disease
CD	Crohn’s disease
GWAS	Genome wide association studies
lncRNAs	Long non-coding RNAs
miRs	microRNAS
MS	Multiple sclerosis
NIK	NF-κB-inducing kinase
PS	Psoriatic arthritis
RA	Rheumatoid arthritis
SLE	Systemic lupus erythematosus
SNPs	Single nucleotide polymorphisms
SS	Sjogren’s disease
Th1	T-helper 1 cells
Th2	T-helper 2 cells
Th17	T-helper 17 cells
Tregs	T-regulatory cells
UC	Ulcerative colitis
WES	Whole exome sequencing
